# Assessment of the Safety Signal for the Abuse Potential of Pregabalin and Gabapentin Using the FAERS Database and Big Data Search Analytics

**DOI:** 10.3389/fpsyt.2021.640264

**Published:** 2021-05-20

**Authors:** Georgios Papazisis, Dimitrios Spachos, Spyridon Siafis, Niki Pandria, Eleni Deligianni, Ioannis Tsakiridis, Antonios Goulas

**Affiliations:** ^1^Department of Clinical Pharmacology, School of Medicine, Aristotle University of Thessaloniki, Thessaloniki, Greece; ^2^Laboratory of Medical Physics, School of Medicine, Aristotle University of Thessaloniki, Thessaloniki, Greece; ^3^Department of Obstetrics and Gynaecology, School of Medicine, Aristotle University of Thessaloniki, Thessaloniki, Greece; ^4^First Department of Clinical Pharmacology, School of Medicine, Aristotle University of Thessaloniki, Thessaloniki, Greece

**Keywords:** pregabalin, gabapentin, big data, Google search analytics, disproportionality analysis, abuse potential, safety signal, FAERS database

## Abstract

**Introduction:** The latest decade, an emerging issue has been the abuse potential of the gabapentinoids pregabalin and gabapentin. The aim of our study was to assess this safety signal combining two different methods of surveillance: search analytics big data and the FDA spontaneous reporting system database.

**Methods:** Analysis of big data and the FAERS was used to detect pregabalin's and gabapentin's abuse potential in comparison with two controls, clonazepam and levetiracetam, and further, the correlation between these domains was investigated. Data from the United States between 2007 and 2020Q2 were analyzed.

**Results:** The FAERS analysis revealed the following pattern of signals: clonazepam > pregabalin ≥ gabapentin > levetiracetam, for both the primary term “drug abuse and dependence” and the secondary terms (withdrawal, tolerance, overdose). The Google domain pattern was slightly different: clonazepam ≥ gabapentin ≥ pregabalin≥ levetiracetam. A monotonic correlation was found between FAERS and Google searches for gabapentin (*r* = 0.558; *p* < 0.001), pregabalin (*r* = 0.587; *p* < 0.001), and clonazepam (*r* = 0.295; *p* = 0.030).

**Conclusion:** Our results revealed that there is preliminary evidence of a safety signal for the abuse potential of pregabalin and gabapentin. Analysis of the FAERS database, supplemented by big data search analytics, suggests that there is potential of using these methods as a supplementary tool to detect drug abuse-related safety signals in pharmacovigilance.

## Introduction

Gabapentinoids (pregabalin and gabapentin) are a class of drugs that have been widely used-prescribed for neuropathic pain, epilepsy, anxiety, and other psychiatric disorders, while pregabalin showed promise as a treatment for alcohol dependence (1, 2). Gabapentin and pregabalin have a similar structure and are derivatives of the inhibitory neurotransmitter GABA. Their proposed mechanism of action is the inhibition of calcium currents via high-voltage-activated channels containing the a2d-1 subunit (3). Since their first approval, both gabapentinoids are widely prescribed medications in the United States (4, 5).

The latest decade, an emerging issue has been the abuse potential of both pregabalin and gabapentin. An increase in non-medical use of gabapentinoids for recreational purposes has been reported, especially in Europe (6, 7). Higher doses of gabapentinoids use have been characterized by causing euphoria effects and a range of experiences such as relaxation, improved sociability, and sedative and psychedelic-like effects (8). From the EudraVigilance database review on gabapentinoids, fatalities were also reported associated with pregabalin and gabapentin use and in most of the cases in combination with opioids (9). Pharmacovigilance data from the Food and Drug Administration Adverse Event Reporting System (FAERS) have shown adverse drug events from gabapentinoid abuse with a higher prevalence in young and male individuals (10). Both pregabalin and gabapentin from 1st April 2019 have been classified as Schedule 3 controlled drugs under the Misuse of Drugs Regulations 2001, and Class C of the Misuse of Drugs Act 1971 in the UK. On the other hand, in the US, pregabalin is a Schedule 5 controlled substance while gabapentin is a controlled substance only in some States. In Australia, pregabalin and gabapentin are classified as Schedule 4 (prescription only) medications; therefore there are no special control measures on supply or possession yet (11).

Considering the abovementioned data on the relative well-established abuse potential profile of the gabapentinoids, the aim of our study was (i). to detect pregabalin's and gabapentin's abuse potential in comparison with two controls, clonazepam and levetiracetam and (ii). to investigate the correlation between the search analytics and the FAERS domain. Our group has recently published the methodology of combining these pharmacovigilance domains in order to detect safety signals (12, 13).

## Methods

### Data Sources

Following the methodology of our previous analysis that investigated mirtazapine's abuse liability (12), herein, we investigated the abuse liability of the gabapentinoids combining pharmacovigilance and search analytics data from the United States between 2007 and 2020Q2. Clonazepam, a frequently used benzodiazepine with a well-known abuse potential profile, was used as a positive control (12, 14), while levetiracetam (a well-known antiepileptic with a low abuse potential) (15) served as negative control.

### FAERS

The pharmacovigilance database of the FAERS consists of individual safety reports originated mainly from the United States. The structure and data mining algorithms of FAERS have been described elsewhere (16). Briefly, reports can be submitted by patients, the pharmaceutical industry, and healthcare professionals, while adverse events are classified with MedDRA terminology (16, 17). The freely available pharmacovigilance tool OpenVigil-2.1-MedDRA (available at http://openvigil.sourceforge.net/) was used in order to access cleaned FAERS data, by removing duplicates and normalizing drug names to the generic name of the drug (18). Similar to our previous analysis, higher level terms were used, whenever possible, to classify reports with drug-abuse-related adverse events (12). The narrow scope of the Standardized MedDRA Query (SMQ) “drug abuse and dependence” was used as the primary term, and other terms related to drug abuse, including overdose, tolerance, withdrawal, and euphoria-related events, were used as secondary terms (Table 1) (12, 19). Disproportionality analysis was conducted for the aggregated period of 2007–2020Q2 for both the primary and secondary terms, while correlation analyses were conducted using quarterly data of the primary term.

**Table 1:** Drug names and drug abuse-related terms.

	**Google**	**FAERS**
Drugs	• Clonazepam, Klonopin • Gabapentin, Neurontin • Pregabalin, Lyrica • Levetiracetam, Kepra	• Clonazepam • Gabapentin • Pregabalin • Levetiracetam
Drug abuse-related terms	{Abuse, dependence}	Drug abuse and dependence (SMQ narrow scope)
	{Withdrawal}	Drug withdrawal (SMQ narrow scope)
	{Overdose}	Tolerance [drug tolerance (PT) and drug tolerance increased (PT)]
	{Tolerance}	Overdose [overdose (PT) and intentional overdose (PT)]
	{High}	Euphoria [euphoric mood (PT), feeling abnormal (PT), feeling drunk (PT), feeling of relaxation (PT), dizziness (PT), thinking abnormal (PT), hallucination (PT), inappropriate affect (PT)]

*In the FAERS database, the drugs are registered with their generic names; in Google search analytics, a brand name was also used. Drug-abuse-related MedDRA terms were selected in FAERS, and similar abuse-related search terms in the search analytics domain (SMQ, standardized MedDRA query; PT, preferred term)*.

### Google Analytics

The Google search engine receives more than 5 billion of queries per day (20). Although it does not provide detailed analytics, some indicators, such as the interest over time, are publicly accessible. Usually, search queries contain terms related to the generic and brand names of the drug, combined together with some additional terms (e.g., “Can you get high of…?”). We combined analytics data retrieved using both the generic name and a common brand name of each drug (Table 1).

An important aspect for retrieving analytics data from the Google search engine is the context. We can define the search context by limiting the returned results per category. The widest category is the “general search term,” where Google returns analytics from searches in all categories. However, since we were studying a very specific area of interest, we could also restrict our results in a more specific category (e.g., “medication”). Google is using search semantics to classify each search query and is expected that the more specific category will provide more accurate results. However, depending on the search popularity of some terms, there may not be enough results inside the category context, because Google returns only results that can be considered as big data volumes. In our study, we used only the “prescription drug” category for the extraction of our data.

Next, we defined a set of six abuse-related search terms, similar to the MedDRA abuse-related terms: {“abuse,” “dependence,” “overdose,” “withdrawal,” “tolerance,” and “high”}. Table 1 indicates the relationship of the terms between the FAERS and the Google domains. We used the term “high” as the corresponding term of “euphoria,” as the second did not have enough data.

By default, Google does not return results for searches with terms and queries made by a few people. Moreover special characters (i.e., queries with apostrophes) were filtered—this is a way of normalization that is also made by default. It is also important that Google's tools eliminate repeated searches from the same person over a short period of time. We identified queries containing combinations of the drug names and the abuse-related terms from the set we defined in a previous step. Finally, we filtered the results manually, by dropping out queries unrelated to abuse. For example, while the search query “clonazepam and high blood pressure” contains both the terms “clonazepam” and “high,” it is not related to abuse. Instead, the query “can you get high of pregabalin” is related to abuse and, thus, included to our search results.

### Statistical Analysis

The search interest over time is measured by the search popularity score (SPS) in the Google domain. We used the SPS score to collect metrics related to abuse liability. In the FAERS domain, we used the reporting odds ratio (ROR) for abuse-related adverse events. This methodology of analysis was recently published from our group (12).

### Search Interest Over Time

Google reports top searches for every search query. These are terms (queries) that are most frequently searched with the main term in the same search session and within the selected category, country, or region (21).

The most popular queries are sorted by SPS. The value of SPS is between 0 and 100. The most popular term (in our case the main drug name, e.g., “Lyrica”) has a normalized score of 100, which is the maximum score. All other queries have a score under this value. This indicator represents the total number of searches divided by the total number of related searches on the specific country or region at the given time range. This is the default method used by Google in a tool called “Google Trends,” to compare relative popularity between topics. For example, an SPS of 50 is assigned to a query that has been searched half as often as the top query. Queries with a search rate <1% are not reported and are signed with a 0 SPS which is neither a percentage value nor an absolute value of searches. Combining more than one term or queries, the value can be above 100. Considering the large number of queries, we can safely assume that all referred statistics come from big data volumes.

We obtained the monthly SPS for all abuse-related terms for each drug. We developed timelines representing the cumulative search interest over time for the abuse-related terms beginning at 2007Q1 and ending at 2020Q2.

### Disproportionality Analysis

Disproportionality analysis was conducted to investigate the association between abuse-related events and the tested drugs in comparison to all other drugs and all other events in the FAERS database. The reporting odds ratio (ROR) was used to quantify this association, and a larger ROR demonstrates a more frequent co-reporting of the tested drug and the selected term as well as a stronger safety signal. We detected safety signals when the number of reports with the combination of the tested drug and selected event was >3 and the lower boundary of the 95% confidence interval of ROR was >1 (16). The disproportionality analysis and RORs were calculated using the OpenVigil2.1-MedDRA (18).

### Correlation Between FAERS and Search Analytics Domains

A correlation coefficient is a statistical metric that measures the probability of two variables to change together. It describes both the strength and the direction of the relationship. The Pearson correlation coefficient is the most well-known metric, which evaluates the linear relationship between two variables. The Spearman correlation coefficient evaluates the monotonic relationship between two continuous or ordinal variables. The difference is that, in a monotonic relationship, the variables tend to change in the same direction, increasing or decreasing their values, but not necessarily at a constant rate, as in a linear relationship. Unlike Pearson's correlation, Spearman's method does not require normality of the variables and, thus, it is a non-parametric statistic.

## Results

### Google Search Analytics

**Figure 1:**
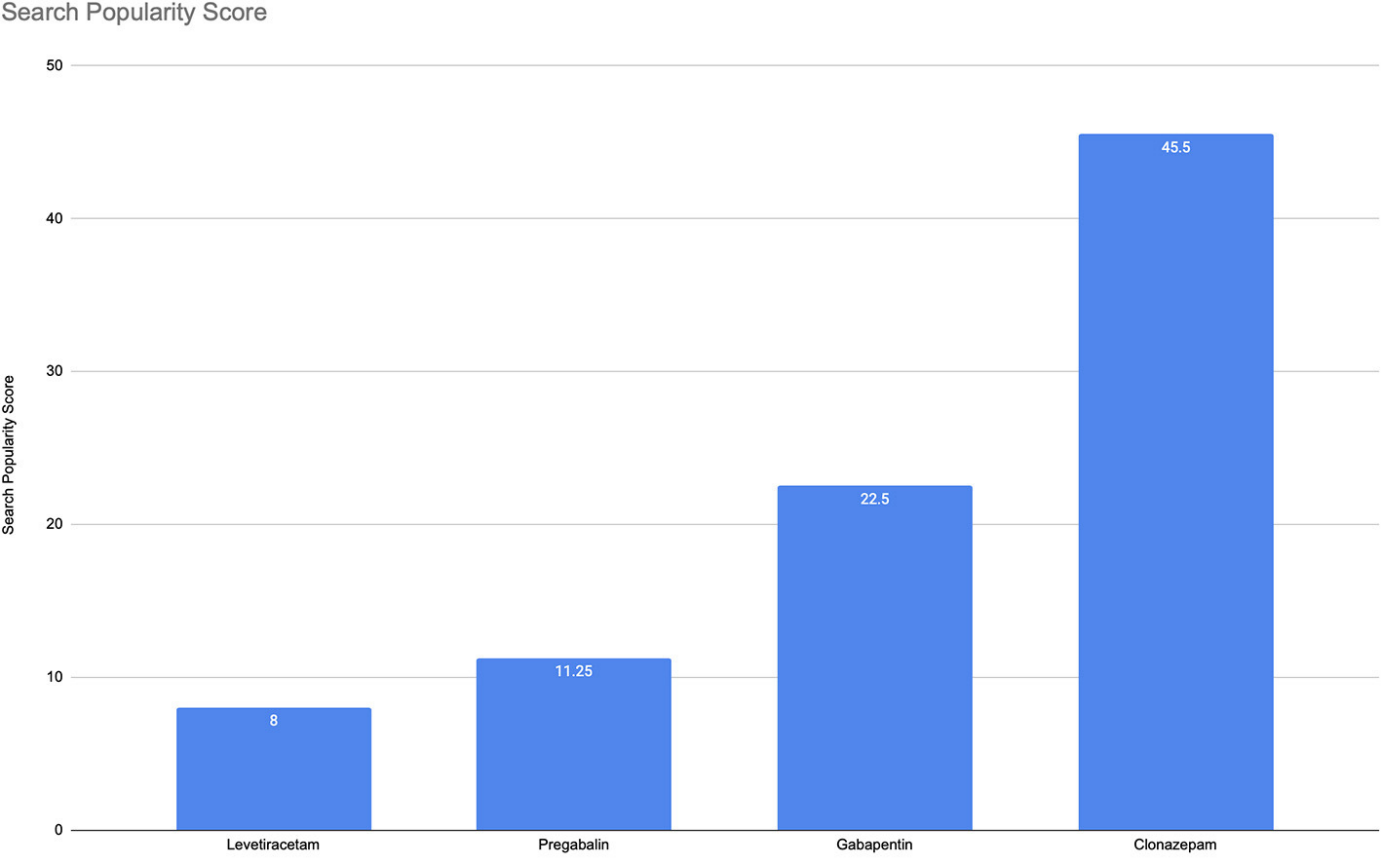
Search Popularity Score (SPS) in the Google analytics domain for the four drugs.

According to the analysis for the cumulative period, the overall abuse-related terms had an average SPS of 8 for Levetiracetam, 11.25 for pregabalin, 22.5 for gabapentin, and 45.5 for Clonazepam (Figure 1). Considering that Google is receiving billion queries per day, even low values of SPS in the given time range represent millions of queries about a topic (22). A non-formal interpretation of these numbers could be as follows: e.g., for pregabalin, for every 100 search queries related to pregabalin, there are 11.25 more queries (on top of the 100) related to pregabalin and abuse related terms.

**Figure 2:**
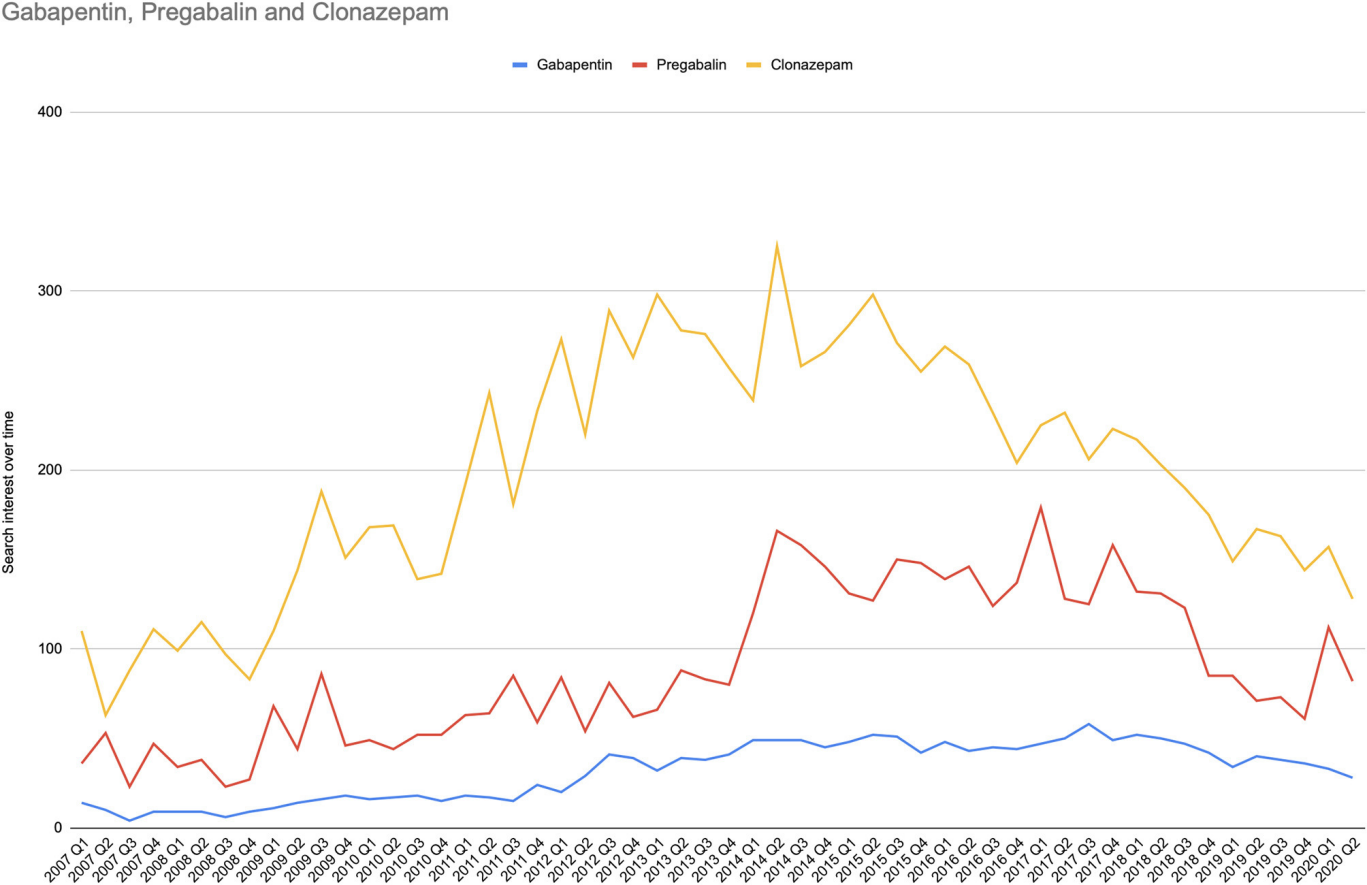
Search interest over time for abuse-related terms in the search analytics domain. For the period 2007Q1 to 2020Q2, the search interest over time for abuse-related terms is represented in timelines for each drug and is expressed as quarterly relative search volume for overall abused-linked terms.

Figure 2 shows the search interest over time for pregabalin, gabapentin, and clonazepam. The search volume for levetiracetam was significantly low, and thus, there were not enough data to be reported by the Google engine. While this may sound as a serious limiting condition, instead it ensures that the reported data are accurate and cannot be affected or modified by a small number of people who perform search queries producing “fake” trends.

The median values of search analytics over time were 82.5, IQR [53.25, 128] for pregabalin, 37, IQR [16.25, 47] for gabapentin, and 203.5, IQR [145.25, 258] for clonazepam.

### Disproportionality Analysis

**Figure 3:**
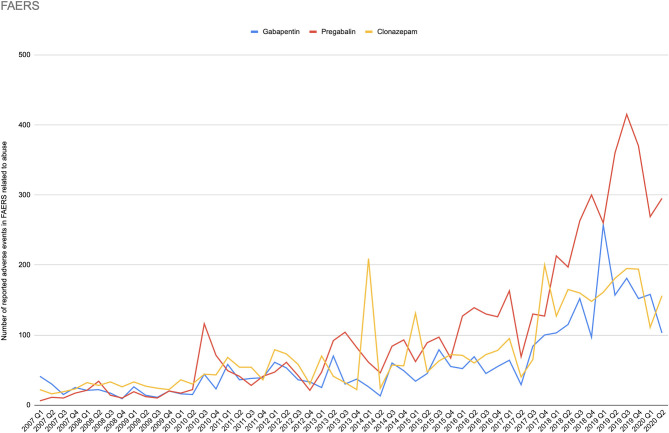
Number of reported adverse events in the FAERS database related to abuse.

During the period of 2007–2020Q2, there were in total 7430750 reports submitted in FAERS. The total number of reports (N) was larger for pregabalin (*N* = 107,905) and gabapentin (*N* = 102,386), and about half for each of the controls, clonazepam (*N* = 55,856), and levetiracetam (*N* = 43,842). For the primary term “drug abuse and dependence” (*N* = 118,980), safety signals were identified for both gabapentinoids (pregabalin: ROR 2.78 95% CI [2.70–2.86]; gabapentin ROR 1.83 95% CI [1.76–1.90]), while the positive control clonazepam had the largest signal (ROR 4.47 95% CI [4.32–4.62]), and the negative control levetiracetam had a very weak signal (ROR 1.10 95% CI [1.02–1.18]). The secondary terms followed the same pattern of signals (clonazepam > pregabalin ≥ gabapentin > levetiracetam), except for euphoria-related terms, for which pregabalin had the largest ROR and overdose-related terms, for which the gabapentinoids and levetiracetam demonstrated similar signals (Table 2). Figure 3 shows the number of reported adverse events related to abuse terms in the FAERS database.

**Table 2:** Number of reports and ROR & 95% CI related to drug abuse per drug.

	**Pregabalin (*N =* 107,905)**	**Gabapentin (*N =* 102,386)**	**Levetiracetam (*N =* 43,842)**	**Clonazepam (*N =* 55,856)**
Drug abuse and dependence (*N =* 118,980)	ROR 2.78 95% CI [2.70–2.86]; *N =* 4,558	ROR 1.83 95% CI [1.76–1.90]; *N =* 2,924	ROR 1.10 95 %CI [1.02–1.18]; *N =* 767	ROR 4.47 95% CI [4.32–4.62]; *N =* 3,700
Drug withdrawal (*N =* 28,149)	ROR 3.76 95% CI [3.56–3.96]; *N =* 1,463	ROR 2.09 95% CI [1.95–2.25]; *N =* 796	ROR 1.54 95 % CI [1.36–1.74]; *N =* 254	ROR 4.81 95% CI [4.51–5.13]; *N =* 976
Overdose (*N =* 85,274)	ROR 1.69 95% CI [1.61–1.76]; *N =* 2,053	ROR 1.65 95% CI [1.57–1.72]; *N =* 1,904	ROR 1.98 95% CI [1.86–2.11]; *N =* 979	ROR 4.29 95% CI [4.12–4.46]; *N =* 2,588
Drug tolerance (*N =* 1,965)	ROR 4.73 95% CI [3.96–5.66]; *N =* 128	ROR 3.76 95% CI [3.07–4.61]; *N =* 98	ROR 0.78 95% CI [0.40–1.49]; *N =* 9	ROR 6.94 95% CI [5.66–8.51]; *N =* 98
Euphoria-related events (*N =* 280,097)	ROR 2.87 95% CI [2.81–2.93]; *N =* 10,664	ROR 2.09 95% CI [2.04–2.14]; *N =* 7,644	ROR 1.27 95% CI [1.22–1.33]; *N =* 2,077	ROR 2.41 95% CI [2.33–2.48]; *N =* 4,765

*Each drug has been compared with all other drugs in the FAERS database. The study population consisted of 6993352 reports*.

### Correlation Between FAERS and Search Analytics Domains

**Figure 4:**
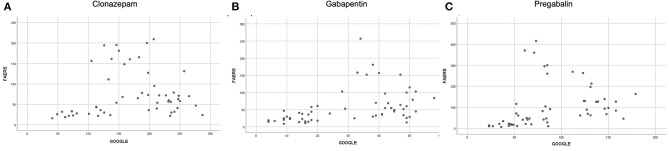
Correlation between FAERS and Google searches for **(A)** clonazepam (*r* = 0.295; *p* = 0.030); **(B)** gabapentin (*r* = 0.558; *p* < 0.001); **(C)** pregabalin (*r* = 0.587; *p* < 0.001).

A monotonic correlation was found between FAERS and Google searches for clonazepam (*r* = 0.295; *p* = 0.030, Figure 4A), gabapentin (*r* = 0.558; *p* < 0.001, Figure 4B), and pregabalin (*r* = 0.587; *p* < 0.001, Figure 4C). Since Google reports only volumes with a significant number of searches, which can be considered as big data volumes, we were not able to collect the amount of data required for analysis for levetiracetam.

## Discussion

Based on extensive literature search, this is the first study investigating the abuse potential of pregabalin and gabapentin using two different pharmacovigilance methods: disproportionality analysis in the FAERS and Google search analytics. A positive control and a negative control were used, the benzodiazepine clonazepam, with a well-known abuse profile and the antiepileptic levetiracetam, with a previously unreported abuse potential, respectively.

### Signals in the FAERS Database

Our disproportionality analysis of the FAERS revealed the following pattern of signals: clonazepam > pregabalin ≥ gabapentin > levetiracetam, both for the primary term “drug abuse and dependence” and the secondary terms (withdrawal, tolerance, overdose). Our results confirm previous findings from the pharmacovigilance domain that highlight the abuse potential of pregabalin. According to the review of the EudraVigilance database, adverse drug reactions were more frequently reported for pregabalin use compared to gabapentin (23). Pharmacovigilance data from FAERS have also shown adverse drug events from pregabalin use and in general gabapentinoid abuse with a prevalence in young and male individuals (10). In contrast, from the EudraVigilance database review, there were adverse drug reaction reports related to abuse/dependence and misuse of pregabalin and gabapentin with a prevalence in female adults (9). The last decade, apart from gabapentinoid abuse there has also been reported extended misuse, with a greater potential of misuse for pregabalin (9). The misuse of pregabalin has been strongly linked to its strong sedative and psychedelic effects. It has been stated that pregabalin misuse is more likely to occur in new users (24). Besides being considered as less powerful than pregabalin, gabapentin misuse was also associated with similar psychedelic effects. A few substances have been reported for misuse in combination with gabapentin, such as cannabis, alcohol, selective serotonin reuptake inhibitors (SSRIs), LSD, amphetamine, and gamma-hydroxybutyrate (GHB) (8, 25). There is agreement from other studies that the majority of individuals that have been reported for pregabalin abuse have a history of other substance and medication abuse as well (11, 26). The differences in the pharmacokinetic and pharmacodynamic profile of the gabapentinoids should be carefully examined in order to understand pregabalin's higher abuse potential compared to gabapentin (11, 25).

### Signals in the Google Analytics Domain

The Google search analytics data are big data. Their volume, velocity, and variety are far beyond any other dataset of collected data, such as the adverse event reports. While they cannot be considered as a safe source for safety signals, their recognition of the potential is rising (27), and their use in pharmacovigilance is emerging. A recently published study of the French Addictovigilance Network combined Google Trends with the analysis of the global database of individual case safety reports (VigiBase) (28). Our team has recently published this method of combining different data sources of drug safety surveillance, Google search analytics, and disproportionality analysis of the US FAERS database (12) to detect safety signals. Data from this timeline series from 2004Q1 to 2017Q2 revealed a consistent association of abuse-related searches in the Google search engine with the antidepressant mirtazapine, and a similar pattern of association between abuse-related events and the drug was found in FAERS. The results of this previous study already suggested that search analytics and disproportionality analysis of FAERS may be used combined as a supplementary pharmacovigilance tool. Signals of gabapentinoid abuse found agreed with the signals for the positive and negative control drugs (clonazepam and levetiracetam). The generic pattern for FAERS was clonazepam ≥ pregabalin ≥ gabapentin ≥ levetiracetam. The Google domain pattern was slightly different: clonazepam ≥ gabapentin ≥ pregabalin ≥ levetiracetam. This difference can be explained by the fact that gabapentin was first approved for use in 1993 and in 2018 it was the eleventh most commonly prescribed medication in the United States, with more than 46 million prescriptions in 2018 and an increasing number of prescription over time (5). On the other hand, pregabalin (FDA approved in 2004) had an estimated number of 11.5 million prescriptions in 2018 in the United States being in ranking 70th among the most commonly prescribed medication (4). It should also be noted that disproportionality analysis cannot quantify the true risk, which should also be the case for the Google domain (29).

### Correlation Between the Domains

A significant monotonic correlation was found between FAERS and Google searches for gabapentin (*r* = 0.558; *p* < 0.001), pregabalin (*r* = 0.587; *p* < 0.001), and clonazepam (*r* = 0.295; *p* = 0.030). This relationship between two totally different domains indicates that when one of the values changes in one domain, there is a significant probability to change in the same way in the other domain. Thus, changes of abuse-related searches on Google for pregabalin, gabapentin, or clonazepam are accompanied by analogous changes of abuse-related events in FAERS and vice versa. There is no causality on this fact but, rather, a similar behavior of two data domains. Interestingly, there were not enough big data volumes for levetiracetam to develop the timelines and, thus, no comparison could be made.

### Study Limitations

Our study has some methodological considerations and limitations. Disproportionality analysis cannot differentiate between recreational, self-treatment, or mixed type of abuse; however, it is a suitable tool to quantitate signals of abuse of known and novel psychoactive substances. Further, the causal relationship between drugs and the adverse event (abuse) cannot be verified without a clinically performed causality assessment, while confounders as comorbidity and concomitant drugs cannot also be assessed properly. Regarding search analytics, since Google only reports large datasets, terms such as dependence, tolerance, and misuse have not provided substantial numbers and were not included in the analysis. In addition, the algorithms and their updates utilized by Google to analyze data are not publicly available. Finally, there were not enough data volumes before 2007.

## Conclusion

Concluding, the present study revealed a safety signal for the abuse potential of pregabalin and gabapentin using two different methods of surveillance, the FAERS database analysis and big data search analytics. We suggest that these methods can be used in combination as a supplementary pharmacovigilance tool to detect drug safety signals.

## Data Availability Statement

Publicly available datasets were analyzed in this study. This data can be found at: http://openvigil.sourceforge.net/.

## Author Contributions

GP: project development and manuscript writing and editing. DS and SS: data collection, data analysis, and manuscript drafting. NP and IT: data analysis and manuscript editing. AG: review of the final manuscript. All authors contributed to the article and approved the submitted version.

## Conflict of Interest

The authors declare that the research was conducted in the absence of any commercial or financial relationships that could be construed as a potential conflict of interest.

## References

[1] Di Nicola M Martinotti G Tedeschi D Frustaci A Mazza M Sarchiapone M. Pregabalin in outpatient detoxification of subjects with mild-to-moderate alcohol withdrawal syndrome. Hum Psychopharmacol. (2010) 25:268–75. 10.1002/hup.1098 20373479

[2] Martinotti G Nicola M Tedeschi D Mazza M Janiri L Bria P. Efficacy and safety of pregabalin in alcohol dependence. Adv Ther. (2008) 25:608–18. 10.1007/s12325-008-0066-2 18553183

[3] Tzellos TG Papazisis G Toulis KA Sardeli C Kouvelas D. A2δ ligands gabapentin and pregabalin: future implications in daily clinical practice. Hippokratia. (2010) 14:71–5. 20596259 PMC2895293

[4] ClinCalk. Pregabalin Drug Usage Statistics, United States, 2008 - 2018 (2020).

[5] ClinCalk. Gabapentin Drug Usage Statistics, United States, 2008 - 2018. (2020). Available online at: https://clincalc.com/DrugStats/Drugs/Gabapentin (accessed December 8, 2020).

[6] European Monitoring Centre for Drugs and Drug Addiction. European Drug Report 2014: Trends and Developments (2014).

[7] Martinotti G Lupi M Sarchione F Santacroce R Salone A Berardis D. The potential of pregabalin in neurology, psychiatry and addiction: a qualitative overview. Curr Pharm Des. (2013) 19:6367–74. 10.2174/13816128113199990425 23782139

[8] Schifano F. Misuse and abuse of pregabalin and gabapentin: cause for concern? CNS Drugs. (2014) 28:491–6. 10.1007/s40263-014-0164-4 24760436

[9] Chiappini S Schifano F. A decade of gabapentinoid misuse: an analysis of the European Medicines Agency's ‘suspected adverse drug reactions’ database. CNS Drugs. (2016) 30:647–54. 10.1007/s40263-016-0359-y 27312320

[10] Evoy KE Covvey JR Peckham AM Ochs L Hultgren KE. Reports of gabapentin and pregabalin abuse, misuse, dependence, or overdose: an analysis of the Food And Drug Administration Adverse Events Reporting System (FAERS). Res Soc Adm Pharm. (2019) 15:953–8. 10.1016/j.sapharm.2018.06.018 31303196

[11] Papazisis G Tzachanis D. Pregabalin's abuse potential: a mini review focusing on the pharmacological profile. Int J Clin Pharmacol Ther. (2014) 52:709–16. 10.5414/CP202118 24849194

[12] Spachos D Siafis S Bamidis P Kouvelas D Papazisis G. Combining big data search analytics and the FDA Adverse Event Reporting System database to detect a potential safety signal of mirtazapine abuse. Health Informatics J. (2020) 26:2265–79. 10.1177/1460458219901232 32026758

[13] Spachos D Siafis S Bamidis P Kouvelas D Papazisis G. Detecting a potential signal of quetiapine abuse using the faers database and big data search analytics. Drug Saf. (2018) 41:1236.

[14] Bossard JB Ponté C Dupouy J Lapeyre-Mestre M Jouanjus E. Disproportionality analysis for the assessment of abuse and dependence potential of pregabalin in the French pharmacovigilance database. Clin Drug Investig. (2016) 36:735–42. 10.1007/s40261-016-0421-z 27300651

[15] Schoedel KA Stockis A Sellers EM. Human abuse potential of brivaracetam in healthy recreational central nervous system depressant users. Epilepsy Behav. (2018) 78:194–201. 10.1016/j.yebeh.2017.09.008 29153631

[16] Sakaeda T Tamon A Kadoyama K Okuno Y. Data mining of the public version of the FDA adverse event reporting system. Int J Med Sci. (2013) 10:796–803. 10.7150/ijms.6048 23794943 PMC3689877

[17] Harrison J Mozzicato P. MedDRA^®^: the tale of a terminology: side effects of drugs essay. Side Effects Drugs Ann. (2009) 31: xxxiii–xli. 10.1016/S0378-6080(09)03160-2

[18] Böhm R Von Hehn L Herdegen T Klein HJ Bruhn O Petri H. OpenVigil FDA - Inspection of U.S. American adverse drug events pharmacovigilance data and novel clinical applications. PLoS ONE. (2016) 11: e0157753. 10.1371/journal.pone.0157753 27326858 PMC4915658

[19] Brooks SK Webster RK Smith LE Woodland L Wessely S Greenberg N. The psychological impact of quarantine and how to reduce it: rapid review of the evidence. Lancet. (2020) 395:912–20. 10.1016/S0140-6736(20)30460-8 32112714 PMC7158942

[20] Berners-Lee T. Google Search Statistics. Internet Live Stats. (2017). Available online at: http://www.internetlivestats.com/google-search-statistics (accessed December 10, 2020).

[21] support.google.com. The Homepage Explained Title. Available online at: https://support.google.com/trends/answer/6248105?hl=en&ref_topic=6248052

[22] Van Puijenbroek EP Bate A Leufkens HGM Lindquist M Orre R Egberts ACG. A comparison of measures of disproportionality for signal detection is spontaneous reporting systems for adverse drug reactions. Pharmacoepidemiol Drug Saf. (2002) 11:3–10. 10.1002/pds.668 11998548

[23] Schifano F Chiappini S Corkery JM Guirguis A. An insight into Z-drug abuse and dependence: an examination of reports to the European Medicines Agency Database of suspected adverse drug reactions. Int J Neuropsychopharmacol. (2019) 22:270–7. 10.1093/ijnp/pyz007 30722037 PMC6441128

[24] Driot D Jouanjus E Oustric S Dupouy J Lapeyre-Mestre M. Patterns of gabapentin and pregabalin use and misuse: results of a population-based cohort study in France. Br J Clin Pharmacol. (2019) 85:1260–9. 10.1111/bcp.13892 30737829 PMC6533441

[25] Martinotti G Papazisis G Santacroce R Kouvelas D Cinosi E Lupi M di Giannantonio M. Pregabalin abuse and addiction. In: Preedy VR, editor. Neuropathology of Drug Addictions and Substance Misuse. London, UK: Academic Press (2016). p. 945–51. 10.1016/B978-0-12-800634-4.00093-7

[26] Papazisis G Garyfallos G Sardeli C Kouvelas D. Pregabalin abuse after past substanceseeking behavior. Int J Clin Pharmacol Ther. (2013) 51:441–2. 10.5414/CP201881 23547854

[27] Kruse CS Goswamy R Raval Y Marawi S. Challenges and opportunities of big data in health care: a systematic review. JMIR Med Informatics. (2016) 4:e38. 10.2196/medinform.5359 27872036 PMC5138448

[28] Ponté C Pi C Palmaro A Jouanjus E Lapeyre-Mestre M. Early signal of diverted use of tropicamide eye drops in France. Br J Clin Pharmacol. (2017) 83:1791–800. 10.1111/bcp.13272 28239898 PMC5510080

[29] Montastruc JL Sommet A Bagheri H Lapeyre-Mestre M. Benefits and strengths of the disproportionality analysis for identification of adverse drug reactions in a pharmacovigilance database. Br J Clin Pharmacol. (2011) 72:905–8. 10.1111/j.1365-2125.2011.04037.x 21658092 PMC3244636

